# *In silico* structure-based designers of therapeutic targets for diabetes *mellitus* or obesity: A protocol for systematic review

**DOI:** 10.1371/journal.pone.0279039

**Published:** 2022-12-12

**Authors:** Ana Francisca Teixeira Gomes, Wendjilla Fortunato de Medeiros, Gerciane Silva de Oliveira, Isaiane Medeiros, Juliana Kelly da Silva Maia, Ingrid Wilza Leal Bezerra, Grasiela Piuvezam, Ana Heloneida de Araújo Morais

**Affiliations:** 1 Nutrition Postgraduate Program, Center for Health Sciences, Federal University of Rio Grande do Norte, Natal, RN, Brazil; 2 Biochemistry and Molecular Biology Postgraduate Program, Biosciences Center, Federal University of Rio Grande do Norte, Natal, Brazil; 3 Department of Nutrition, Center for Health Sciences, Federal University of Rio Grande do Norte, Natal, RN, Brazil; 4 Public Health Postgraduate Program, Center for Health Sciences, Federal University of Rio Grande do Norte, Natal, Brazil; 5 Department of Public Health, Center for Health Sciences, Federal University of Rio Grande do Norte, Natal, Brazil; Xiamen University - Malaysia Campus: Xiamen University - Malaysia, MALAYSIA

## Abstract

Obesity is a significant risk factor for several chronic non-communicable diseases, being closely related to Diabetes *Mellitus*. Computer modeling techniques favor the understanding of interaction mechanisms between specific targets and substances of interest, optimizing drug development. In this article, the protocol of two protocols of systematic reviews are described for identifying therapeutic targets and models for treating obesity or diabetes *mellitus* investigated *in silico*. The protocol is by the guidelines from the Preferred Reporting Items for Systematic Reviews and Meta-Analyzes Protocols (PRISMA-P) and was published in the International Prospective Register of Systematic Reviews database (PROSPERO: CRD42022353808). Search strategies will be developed based on the combination of descriptors and executed in the following databases: PubMed; ScienceDirect; Scopus; Web of Science; Virtual Health Library; EMBASE. Only original *in silico* studies with molecular dynamics, molecular docking, or both will be inserted. Two trained researchers will independently select the articles, extract the data, and assess the risk of bias. The quality will be assessed through an adapted version of the Strengthening the Reporting of Empirical Simulation Studies (STRESS) and the risk of bias using a checklist obtained from separate literature sources. The implementation of this protocol will result in the elaboration of two systematic reviews identifying the therapeutic targets for treating obesity (review 1) or diabetes *mellitus* (review 2) used in computer simulation studies and their models. The systematization of knowledge about these treatment targets and their *in silico* structures is fundamental, primarily because computer simulation contributes to more accurate planning of future either *in vitro* or *in vivo* studies. Therefore, the reviews developed from this protocol will guide decision-making regarding the choice of targets/models in future research focused on therapeutics of obesity or Diabetes *Mellitus* contributing to mitigate of factors such as costs, time, and necessity of *in vitro* and/or *in vivo* assays.

## Introduction

Rising numbers of obesity and diabetes cases have provided a favorable backdrop for the growth of a new epidemic named “Diabesity” [1]. The term aims to define the coexistence of these two comorbidities in the same individual [1, 2]. This increase highlights the undeniable limitations regarding managing both conditions and the crucial importance of the global effort in the search for new treatments [1]. The urgency is widely due to the economic and public health burden [3, 4].

In obesity, changes in the organization of adipose tissue trigger a state of low-grade systemic inflammation and metabolic complications, leading to a greater predisposition to Noncommunicable diseases (NCDs) like diabetes *mellitus* (DM). These diseases contribute to the reduction in the quality of life and mortality [5]. It is estimated that the number of people with obesity and DM increased worldwide, and projections point to a growing trend in the coming years [6, 7].

Several strategies are adopted for treating obesity and DM, such as lifestyle changes, physical exercise, pharmacological therapy, and even natural alternatives such as bioactive molecules [4, 8, 9]. Therapeutic agents, whether antiobesogenic or antidiabetic, can be selected according to biological effects caused by the interaction with some molecular target able to modify the course of the disease. This target-based approach is one form of drug discovery [10].

Studies by computer simulations or *in silico* studies can enhance understanding of the mechanisms of action and interaction between therapeutic targets and agents. The *in silico* techniques used in bioinformatics, such as molecular docking or molecular dynamics, are the most common methods used in Structure-based drug design (SBDD) being one general types of computer-aided drug design (CADD), that allow the development of drugs and the understanding of effects caused by molecules with bioactive properties, in addition to demonstrating the potential side effects and toxicity if these are predisposing factors related to the target or agent [11–13], dispensing the use of laboratory animals (ethical principle of the Replacement, Reduction and Refinement – 3Rs) [14].

Given the importance of *in silico* studies for the scientific community, there is an increasing number of systematic reviews encompassing this approach, allowing a critical evaluation of the results evidenced by several studies on a given topic and the synthesis of the main findings [15]. Several investigations are carried out such as the search for antimicrobial molecules for applications in the treatment of tuberculosis [16], drug repurposing using molecular docking to treat COVID-19 [17], use of *in silico* methods as a screening tool for the search for flavonoid-derived drugs [18], among others.

Thus, the systematization of potential therapeutic targets, used by *in silico* studies for treating obesity and DM, will drive future *in vitro*, *in vivo*, and preclinical trials to validate of the findings demonstrated through bioinformatics. Moreover, will contribute to researchers in identifying new ligands or targets and will be fundamental for developingt drugs or natural agents with the potential to control these diseases. Therefore, this article aims to detail the protocol of two systematic reviews that will be developed with *in silico* studies to identify therapeutic targets for treating obesity and DM.

## Methods/Design

### Registration protocol

This systematic review protocol was designed according to the recommendations of the Preferred Report Items for Systematic Reviews and Meta-analyses (PRISMA-P) [19]. The protocol was registered on the Prospective International Systematic Reviews platform (PROSPERO), (registration number CRD42022353808), available in: https://www.crd.york.ac.uk/prospero/display_record.php?ID=CRD42022353808.

### Review question

The present protocol was conducted to answer the following questions that will correspond to two distinct systematic reviews:

- What therapeutic targets have been used in *in silico* analysis for the treatment of obesity?
- What therapeutic targets have been used in *in silico* analysis for the treatment of diabetes *mellitus*?

Such questions were established according to the PECo strategy (P, problem; E, exposure; Co, context) (Tables 1 and 2).

**Table 1:** Research question structure (What therapeutic targets have been used in *in silico* analysis for the treatment of obesity?) according to the PECo strategy for the systematic review.

Description	Abbreviation	Elements
**Problem**	P	Therapeutic targets used in the treatment of obesity
**Exposure**	E	Obesity
**Context**	Co	*In silico* studies with molecular dynamics or molecular docking

PECo (P, problem; E, exposure; Co, context).

**Table 2:** Research question structure (What therapeutic targets have been used in *in silico* analysis for the treatment of diabetes *mellitus*?) according to the PECo strategy for the systematic review.

Description	Abbreviation	Elements
**Problem**	P	Therapeutic targets used in the treatment of Diabetes *Mellitus*
**Exposure**	E	Diabetes *Mellitus*
**Context**	Co	*In silico* studies with molecular dynamics or molecular docking

PECo (P, problem; E, exposure; Co, context).

### Eligibility criteria

#### Problem

Studies on anti-obesogenic or anti-diabetic therapeutic targets will be included. Studies on therapeutic targets used in treating other comorbidities will be excluded.

#### Exposure

Studies regarding obesity or diabetes *mellitus* will be included. Studies addressing other comorbidities will not be included.

#### Context

Only the original *in silico* studies with molecular dynamics, molecular docking, or both will be inserted. Studies exclusively *in vivo*, *in vitro* and other types of *in silico* that do not correspond to molecular dynamics or molecular docking, will not be included. In addition, preprint studies, review articles, theses, dissertations, letters, conference abstracts, and gray literature will be excluded.

### Information sources and bibliographic research

Two systematic reviews will be carried out following the protocol registered on PROSPERO, one related to therapeutic targets used in the *in silico* studies for treating obesity and the other for treating Diabetes *Mellitus*.

A comprehensive search will be elaborated from combinations of MESH and EMTREE terms combinations using Boolean operators (AND and OR) on databases, without time or language restrictions. The searches will be conducted in the following electronic bibliographic databases: PubMed, ScienceDirect, Scopus, Web of Science, Virtual Health Library, EMBASE. Additionally, a manual search will be carried out to insert articles that may not have been found in the above databases. The search equation for the systematic review will be defined considering the items of the PECo strategy (Tables 3 and 4). Adjustments may be necessary for the search strategy, considering the characteristics of the electronic databases, and terms may be included or changed.

**Table 3:** Search strategy for the Pubmed database to recover articles to answer the systematic review’s question: What therapeutic targets have been used in *in silico* analysis for the treatment of obesity?.

Terms	
Problem	“therapeutic target” OR target OR treatment
Exposure	obesity
Context	(“in silico” OR “computer simulation”) AND (“molecular dynamics simulation” OR “molecular dynamics” OR “molecular docking simulation” OR “molecular docking”)

**Table 4:** Search strategy for the Pubmed database to recover to answer the systematic review’s question: What therapeutic targets have been used in *in silico* analysis for the treatment of diabetes *mellitus*?.

Terms	
Problem	“therapeutic target” OR target OR treatment
Exposure	“Diabetes *mellitus*”
Context	(“in silico” OR “computer simulation”) AND (“molecular dynamics simulation” OR “molecular dynamics” OR “molecular docking simulation” OR “molecular docking”)

### Study selection

The works will be read by two reviewers independently. Initially, the titles and abstracts of the works will be read. Subsequently, the selected works will be read in full. Disagreements will be resolved by a third reviewer.

**Fig 1:**
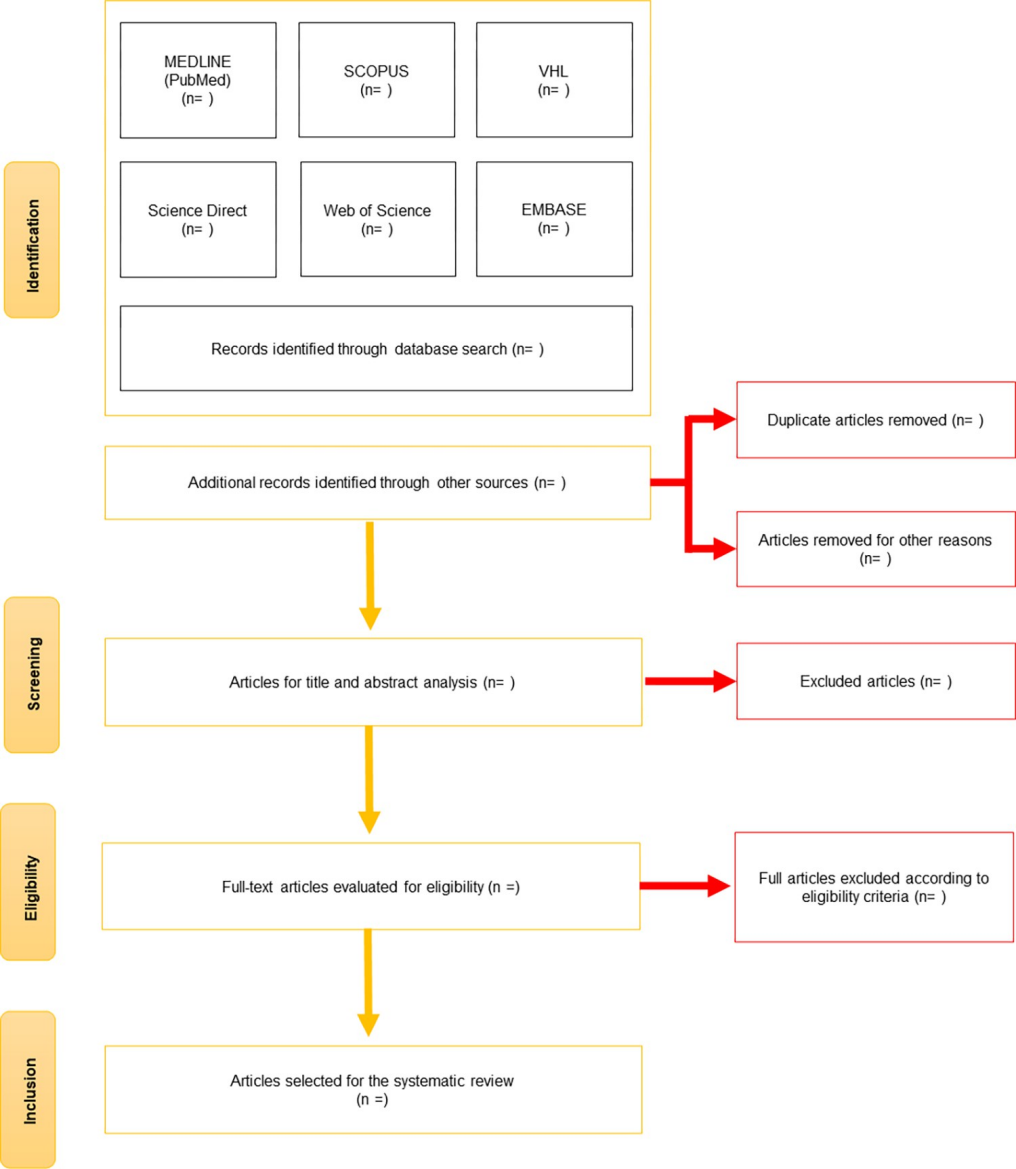
Article selection flowchart adapted from Preferred Reporting Items for Systematic Reviews (PRISMA-P).

After screening, all articles will be imported into the Rayyan app (version 0.1.0) [20]. Migrating articles to this platform will facilitate the removal of duplicate studies (based on inclusion criteria). The reasons for exclusion from studies will be recorded. The report of selecting or excluding studies will be shown using the PRISMA-P flowchart (Fig 1).

### Data extraction

Data extraction will be performed by two reviewers independently. The following characteristics will be extracted from the selected articles: authors, country, model (*in silico*), technique used (docking and molecular dynamics), docking score, potential energy of interaction, amino acids with greater interaction, and the main outcomes are therapeutic targets, therapeutic agent used, effects *in vitro*/*in vivo*, potential applications, and other timely data. Works not found in their entirety will be requested from their respective authors via e-mail (maximum of two attempts). The articles and the respective data will be tabulated in a predefined table using the Microsoft Excel Program.

### Data analysis and synthesis

Data will be summarized through a narrative approach, and the characteristics of the included studies will be described in tables. The reviews will be structured around the type of therapeutic targets in treating obesity or diabetes investigated through *in silico* simulations.

For both reviews, summaries of the results and protocols used in the studies will be provided. Data will be presented in summary tables and narrative forms to describe the characteristics of the included studies. These data will be given according to the type of therapeutic targets related to treating obesity or diabetes, as well as three-dimensional models of these molecules or compounds used in interactions by computer simulations. The references will be organized using the Mendeley software [21]. Due to the methodology of the evaluated studies, no meta-analysis will be applied.

### Risk of bias

Two reviewers will independently assess the quality of the studies and the risk of bias. A third evaluator will resolve discrepancies. Due to the lack of a standardized tool for this type of study, the modeling quality will be evaluated using the checklist developed based on standardized guidelines for simulation “Strengthening the reporting of empirical simulation studies (STRESS)” [22]. And the risk of bias will be assessed using a checklist previously developed and applied by Taldaev et al. [18]. Training will be carried out with the evaluators to ensure uniformity in applying both tools.

## Discussion

It is intended to elaborate on two systematic literature reviews based on articles whose methodology uses bioinformatics tools. Both reviews will focus on identifying of therapeutic targets studied *in silico*, in parallel, respective three-dimensional models used will be systematized. The first review will identify the therapeutic targets of obesity, while the second will aim to reveal the targets of diabetes.

The knowledge about targets and their structures, as well as the advances in bioinformatics techniques, go beyond theoretical limits and impact public health. The possibilities of success in developing effective therapies for difficult-to-control comorbidities, such as diabetes and obesity, are expanded [23, 24].

The stages of discovery and development of new drugs can take around 10 to 12 years and imply a high financial investment [25]. Among these steps, treatment targets must be previously identified and validated, new therapeutic options discovered and/or optimized and, before the arrival of a new drug on the market, pre-clinical and clinical trials carried out [26].

In this regard, one of the great advantages of computer simulation is to reduce the time for designing a new drug and support the accurate design of future *in vitro* and/or *in vivo* studies (serving as a starting point). It is then possible to accelerate development and simultaneously collaborate with the reduction in the use of animal models and research costs [27].

The requirement to systematize knowledge about therapeutic targets has been gained allies, such as open access targeting platforms, including the Open Targets Platform (OTP), which aims to bring scientific evidence on targets and assist in scientific decision-making [24]. However, implementing a bioinformatics study assessing the interaction between a new substance and a specific target still requires that the structures of the substance and target are theoretically and experimentally elucidated.

The existence of elucidated *in silico* structures has been growing in recent years, collaborating with initial efforts to discover and understand the mechanisms of interaction of these new substances with specific targets [28, 29]. Thus, it is indispensable to conduct review studies that bring information not only about the existence of the target but also enable the execution of computer simulation studies with higher quality.

Bioinformatics techniques, such as virtual screening tools in large libraries of compounds, are already a reality for the pharmaceutical industry, which uses them in researching and developing new drugs for several comorbidities, including obesity and diabetes [30, 31]. Structure-based drug design (SBDD) and ligand-based drug design (LBDD) are the two general types of computer-aided drug design (CADD) that approaches can be used, exploring the main structural and physicochemical properties of ligands and/or targets, which allows an understanding of the potential before performing an *in vitro* test [30]. Among the relevant computational techniques, structure-based virtual screening (SBVS), molecular docking and molecular dynamics (MD) simulations are the most common methods used in SBDD [11–13].

Synthetic peptides are examples of the application of bioinformatics techniques, where the elaboration of the same can be done, aiming at the optimization of the interaction of the substances with the chosen targets. Among the most promising synthetic peptides is the recently approved Tirzepatide (LY3298176) for treating DM, whose anti-obesity and anti-diabetes properties have been studied through clinical trials [32].

Among the possible limitations of the studies used in the construction of the reviews, some points can be listed, such as the low quality of the original works included, the three-dimensional molecular structures available, different software used, and insufficient detailed data on the *in silico* analysis. Despite the limitations, the systematic protocol proposes to bring the molecular targets most used in computational research and their respective molecular structures, contributing to the better quality of further studies.

Thus, the protocol will guide the development of the first systematic reviews gathering the therapeutic targets of obesity or diabetes used in computer simulation studies. The findings of systematic reviews should then guide the design of new studies *in silico*, contribute to the choice of promising molecular targets, and to the selection of the most appropriate three-dimensional structures to be applied. Consequently, contributing to the search and boosting studies with molecules, compounds, or substances that are candidates for future anti-obesity or anti-diabetes therapies with great probability of success.

## Supporting information

S1 Checklist Preferred Report Items for Systematic Reviews and Meta-analyses (PRISMA-P) checklist. (PDF)
